# Prevention and Management of Thromboembolism in Patients with Paroxysmal Nocturnal Hemoglobinuria in Asia: A Narrative Review

**DOI:** 10.3390/ijms26062504

**Published:** 2025-03-11

**Authors:** Yasutaka Ueda, Wen-Chien Chou, Yeow-Tee Goh, Ponlapat Rojnuckarin, Jin Seok Kim, Raymond Siu Ming Wong, Lily Lee Lee Wong, Jun Ho Jang, Tzeon-Jye Chiou, Yuzuru Kanakura, Jong Wook Lee

**Affiliations:** 1 Department of Hematology and Oncology, Graduate School of Medicine, Faculty of Medicine, Osaka University, 1-1 Yamadaoka, Suita 565-0871, Japan; 2 Division of Hematology, Department of Internal Medicine, National Taiwan University Hospital, No. 7, Chung Shan S. Rd. (Zhongshan S. Rd.), Zhongzheng, Taipei City 100225, Taiwan; wchou@ntu.edu.tw; 3 Department of Haematology, Singapore General Hospital, Outram Rd., Singapore 169608, Singapore; goh.yeow.tee@singhealth.com.sg; 4 Department of Medicine, Faculty of Medicine, Chulalongkorn University and King Chulalongkorn Memorial Hospital, 1873 Rama IV Rd., Pathum Wan, Bangkok 10330, Thailand; rojnuckarinp@gmail.com; 5 Excellence Center in Translational Hematology, Chulalongkorn University, 1873 Rama IV Rd., Pathumwan, Bangkok 10330, Thailand; 6 Division of Hematology, Department of Internal Medicine, Yonsei University College of Medicine, Severance Hospital, 50-1 Yonsei-ro, Sinchon-dong, Seodaemun-gu, Seoul 03722, Republic of Korea; hemakim@yuhs.ac; 7 Sir Y.K. Pao Centre for Cancer and Department of Medicine & Therapeutics, Prince of Wales Hospital, The Chinese University of Hong Kong, 30-32 Ngan Shing Street, Sha Tin, Hong Kong SAR, China; raymondwong@cuhk.edu.hk; 8 Queen Elizabeth Hospital, 13a, Jalan Penampang, Kota Kinabalu 88200, Sabah, Malaysia; lilwongll@gmail.com; 9 Division of Hematology-Oncology, Department of Medicine, Samsung Medical Center, Sungkyunkwan University School of Medicine, 2066 Seobu-ro, Suwon 16419, Republic of Korea; jh21.jang@samsung.com; 10 Cancer Center, Division of Hematology and Oncology, Department of Medicine, Taipei Municipal Wanfang Hospital, Taipei Medical University, No. 111, Section 3, Xinglong Rd., Wenshan District, Taipei City 11696, Taiwan; 108178@w.tmu.edu.tw; 11 Department of Hematology, Sumitomo Hospital, 5-chōme-3-20 Nakanoshima, Kita Ward, Osaka 530-0005, Japan; kanakura-yuzuru@sumitomo-hp.or.jp; 12 Division of Hematology-Oncology, Hanyang University Seoul Hospital, 222-1 Wangsimni-ro, Seongdong-gu, Seoul 04763, Republic of Korea; jwlee@catholic.ac.kr

**Keywords:** paroxysmal nocturnal hemoglobinuria, complement inhibitor, thromboembolism, thrombosis, Asia

## Abstract

Thromboembolism (TE) is a major cause of morbidity and mortality in patients with paroxysmal nocturnal hemoglobinuria (PNH). This narrative review summarizes available evidence on TE in Asian patients with PNH and discusses practical considerations and challenges for preventing and managing PNH-associated TE in Asian populations. Evidence suggests that, compared with non-Asians, fewer Asian patients have a history of TE (3.6% vs. 8.9%, *p* < 0.01), receive anticoagulants (8.5% vs. 16.2%, *p* = 0.002), or die from TE (6.9% vs. 43.7%, *p* = 0.000). Independent predictors of TE include lactate dehydrogenase ≥ 1.5 × upper limit of normal, pain, and male sex. Clone size alone does not appear to be a reliable estimate of TE risk. D-dimer levels are a useful marker of hemostatic activation, although they are not specific to PNH. Complement inhibition reduces the incidence of TE, although it does not wholly eliminate TE risk. Eligibility criteria and access to complement inhibitors vary across Asia, with limited availability in some countries. Anticoagulation is required to treat acute TE events and for primary or secondary prophylaxis in selected patients. Physicians and patients must stay alert to the signs and symptoms of TE to ensure prompt and appropriate treatment.

## 1. Introduction

Thrombosis is the most serious complication of paroxysmal nocturnal hemoglobinuria (PNH) and a major cause of disease-related deaths [1]. Thrombosis predominantly affects the venous system, although it can also affect arteries, and often occurs at unusual sites such as the splanchnic circulatory system [2,3]. The incidence of thrombosis in PNH patients has decreased considerably since the introduction of complement 5 (C5) inhibitors (e.g., eculizumab, ravulizumab), but the risk is not eliminated completely [4,5,6]. Evidence suggests that thromboembolism (TE) risk may differ between Asian and non-Asian patients with PNH [7,8,9], underscoring the need for a closer examination of the literature relative to Asian populations. This narrative review summarizes the published literature on TE in Asian patients with PNH and discusses practical considerations and challenges for preventing and managing PNH-associated TE in Asian populations.

## 2. Background

### 2.1. Pathophysiology of PNH

Briefly, PNH arises due to clonal expansion of hematopoietic stem cells that have acquired a somatic mutation in the X-linked phosphatidylinositol glycan class A (*PIGA*) gene. Deficiencies of the GPI-anchored complement regulatory proteins CD55 and CD59 on these hematopoietic cells lead to uncontrolled terminal complement activation and intravascular hemolysis, which is the primary clinical manifestation of PNH and one of the main causes of thrombosis [3,10].

### 2.2. Pathophysiology of TE in PNH

The mechanisms underlying the development of TE in patients with PNH are complex, multifactorial, and not fully understood, but they are known to involve interactions between the complement and coagulation systems [1]. Most mechanisms relate to complement dysfunction and its consequences [11]. The pathological effects of platelet activation, intravascular hemolysis, endothelial cell injury, and neutrophil/monocyte activation have also been implicated [11,12].

In PNH, a deficiency of CD59 on platelets renders them susceptible to complement-mediated activation and results in the production of prothrombotic platelet-derived microparticles [11,12,13]. Recent work has shown that membrane attack complex-mediated release of intracellular ADP is the main driver of complement-mediated platelet activation [14]. A positive association between larger PNH clone size and increased procoagulant factor activity has been demonstrated [15]. Intravascular hemolysis leads to an excess of free hemoglobin, which contributes to the depletion of nitric oxide implicated in platelet activation and adhesion [1,16]. Free hemoglobin also has a direct effect on endothelial dysfunction, causes the release of endothelial cell microparticles, produces reactive oxygen species, and increases tissue factor production, contributing to inflammation and coagulation [1,11,12]. Reactive oxygen species are implicated in the formation of neutrophil extracellular traps, which have been linked with venous thrombosis, potentially explaining the high prevalence of TE at atypical venous sites in PNH [11]. Deficiency of other GPI-linked proteins, such as urokinase-type plasminogen activator receptor (involved in clot lysis), heparan sulfate (involved in the binding of antithrombin to endothelial cells), and the GPI anchor for tissue factor pathway inhibitor (a potent anticoagulant protein) may result in impaired fibrinolysis [1,17,18,19]. In addition, C5 activation generates cytokines involved in inflammatory and prothrombotic processes [1,20]. There is also evidence that altered expression of certain genes (e.g., *BMPR2*, *THBD*, *THBS1*, *MUC4*) may increase the risk of TE in PNH patients [21,22,23].

Evaluations of patients with PNH before treatment with eculizumab (including patients with or without a history of TE) revealed increased levels of D-dimers and prothrombin fragment 1 + 2 (reflecting coagulation activation and thrombin generation), vascular cell adhesion molecule, von Willebrand factor and endothelial microparticles (reflecting endothelial cell activation/dysfunction), and interleukin-6 (reflecting inflammation) [24,25]. Increased fibrinogen and thrombin generation in patients with PNH may lead to a prothrombotic clotting phenotype, characterized by faster clot formation and denser clot structure [26].

### 2.3. Role of Complement Inhibition in Preventing and Managing TE

Given that several factors involved in TE formation in patients with PNH relate to the consequences of complement dysfunction, C5 inhibitors have a major role in preventing and managing TE. Several analyses have shown that eculizumab (the first-in-class terminal complement inhibitor) reduces the rate of TE by six- to sevenfold relative to before treatment. A pooled analysis of three eculizumab clinical trials (total patients n = 195) found that the TE rate decreased by 85% during eculizumab treatment compared with that before eculizumab initiation (from 7.37 to 1.07 events/100 patient-years, *p* < 0.001). The effect was sustained during long-term treatment, with an 81.8% reduction in the incidence of TE observed after a median of 30 (maximum 66) months of follow-up, at which point 96.4% of patients remained free of TE [27]. A retrospective study from the UK (n = 79) reported a decrease in the TE rate from 5.6 events/100 patient-years before eculizumab to 0.8 events/100 patient-years during eculizumab treatment (*p* < 0.001) [28]. Analyses of the International PNH Registry also reported a reduction in the risk of TE and major adverse vascular events (MAVE) with eculizumab [29,30]. In the largest of these analyses (n = 4118), among 1613 patients treated with eculizumab, the risk of TE was reduced by 60% (adjusted hazard ratio [aHR] 0.40, 95% confidence interval [CI] 0.26–0.62, *p* < 0.0001) and that of MAVE by 63% (aHR 0.37, 95% CI 0.26–0.54, *p* < 0.0001) compared with the untreated time [30].

Clinical trials of ravulizumab demonstrated its noninferiority to eculizumab in patients with PNH [31,32]. Long-term follow-up of ravulizumab recipients (n = 441) in these pivotal trials showed a durable efficacy, with only 1.4% of patients experiencing MAVE during up to 2 years of follow-up [33]. Patients (including children) receiving eculizumab can be safely and effectively switched to ravulizumab [32,34,35]. Moreover, ravulizumab provides sustained complement inhibition with a less burdensome dosing regimen compared with eculizumab [32,34,35].

Clinical studies that have assessed mechanistic evidence for the effect of C5 inhibition on TE suggest that the antithrombotic effect may be the result of a reduction in thrombin and fibrinogen generation, endothelial activation, and inflammation. Treatment with eculizumab led to rapid and sustained decreases in various markers of coagulation activation and thrombin generation (prothrombin fragment 1 + 2, D-dimers, thrombin–antithrombin complex), fibrinogen generation, reactional fibrinolysis (plasmin–antiplasmin complexes), and endothelial cell activation (tissue plasminogen activator, vascular cell adhesion molecule, von Willebrand factor, tissue factor pathway inhibitor, and tissue factor-bearing microparticles) [24,25,26,36]. Eculizumab also reduced the rate of clot formation and clot density [26] and was associated with a decrease in interleukin-6 (a marker of inflammation) levels [25].

## 3. TE in Asian PNH Patients

### 3.1. Incidence of TE in Asian PNH Patients

Studies in Asian PNH patients (from China, Japan, Korea, and Taiwan) have reported a history of TE in 6–18% of patients [7,37,38,39,40,41], which is broadly similar to the 13.3% rate among International PNH Registry patients (n = 4439) at the time of enrollment (i.e., prior to treatment with eculizumab) [42]. Other studies from Europe and the United States (US) have reported TE rates ranging from 6% to 39% in PNH patients from Western countries [7,28,43,44,45,46].

**Figure 1:**
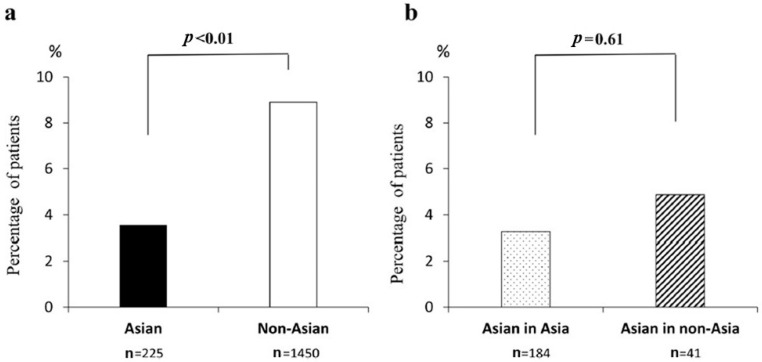
Proportion of Asian and non-Asian patients with thromboembolism at enrollment into the International PNH Registry: (**a**) overall Asian versus non-Asian patients and (**b**) Asian patients enrolled from Asian countries versus Asian patients enrolled from non-Asian countries [9].

A closer examination of TE incidence by population subgroup indicates that thrombosis is less common in Asian than non-Asian patients with PNH. Despite a similar bone marrow status between Asian (n = 246) and non-Asian (n = 1547) subgroups at enrollment in the International PNH Registry, significantly fewer Asian patients had a history of TE at baseline (3.6% vs. 8.9%, *p* < 0.01; Figure 1). The nonsignificant difference in the proportions of Asian patients from Asian (n = 202) and non-Asian (n = 44) countries with TE at baseline (3.3% vs. 4.9%, *p* = 0.61) in this Registry analysis suggests that genetic factors play a stronger role than lifestyle factors in the development of TE [9]. Other studies have reported similar findings. In an epidemiological analysis, significantly fewer Japanese patients than US patients had a history of thrombosis at the time of PNH diagnosis (6.2% [13/209] vs. 19.3% [34/176], *p* < 0.0001), and new thrombotic events were less frequent in Japanese patients during follow-up (4.3% vs. 31.8%, *p* < 0.0001) [7]. A meta-analysis (six retrospective studies, 1665 patients) reported that the incidence rate of TE events was significantly lower in Asian than European/American patients with PNH (11.5% vs. 32.5%, *p* = 0.0000) [8]. Lastly, 6.7% (4/60) of Taiwanese patients in the International PNH Registry had a history of TE compared with 13.5% (502/3710) of patients from the rest of the world, although the difference was not statistically significant (*p* = 0.178) [47]. In pediatric patients with PNH, limited data suggest that the incidence of thrombosis is lower among Asian patients (0–6%) [48,49,50] than non-Asian patients (18–50%) [51,52,53].

The frequency of venous TE was significantly lower in Asian than non-Asian patients in the International PNH Registry (3.1% vs. 7.3%, *p* = 0.02). The frequency of arterial TE was numerically lower for Asian than non-Asian patients, but the difference was not statistically significant (0.4% vs. 2.1%, *p* = 0.09) [9]. In a meta-analysis, abdominal venous thrombosis was found to account for a smaller proportion of TE events in Asian patients than in European/American patients (35.1% vs. 50.5%, *p* = 0.024). In contrast to the International PNH Registry study, however, arterial thrombosis made up a significantly larger proportion of TE events in Asian than non-Asian patients (23.0% vs. 1.1%, *p* = 0.000) [8]. The discord between these two analyses warrants further investigation.

Studies in Asian populations have reported that 1.3–2.1% of PNH patients died from TE events [8,41,54]. Among Asian PNH patients who died from any cause, the proportion of deaths attributed to TE is reported as 6.9–29.6% across studies [7,8,41,55]. Whereas the overall death rate was similar between Asian and European/American patients in a meta-analysis, fewer Asian than non-Asian patients died from TE (6.9% vs. 43.7%, *p* = 0.000) [8]. Similarly, an epidemiological study found that thrombosis was responsible for fewer deaths among Japanese than US patients with PNH (7.9% vs. 42.1%, *p* = 0.0006) [7].

Analyses of the Korean PNH Registry identified TE as an independent risk factor for mortality (odds ratio ~7, *p* < 0.001), increasing mortality by 12- to 14-fold in PNH patients compared with the age- and sex-matched general population [49,56]. Thrombosis was also identified as an independent adverse prognostic factor in Chinese patients with PNH (*p* = 0.0003) [40]. Predictors of TE risk are discussed later in the review.

For reference, a comparison of thrombotic characteristics between Asian and non-Asian patients with PNH is presented in Table 1. Data sources are a meta-analysis of six retrospective studies on clinical characteristics of PNH patients from Asian countries (Japan, Korea, and China) and Western countries (France, the US, and the UK) [8] and an analysis of Asian and non-Asian patients enrolled in the International PNH Registry [9].

### 3.2. Treatment of TE in Asian PNH Patients

Consistent with a lower TE rate among Asian PNH patients, anticoagulants are used less frequently, being administered to 3.3–14.6% of Asian patients as per available published reports [7,9,38,39,47]. In the International PNH Registry, the rate of anticoagulant use was 8.5% in Asian patients and 16.2% in non-Asian patients (*p* = 0.002) [9]. Fewer Asian patients enrolled in the International PNH Registry from Asian than non-Asian countries were treated with anticoagulant therapy (6.7% vs. 17.1%, *p* = 0.03) [9]. Anticoagulant use was found to be lower among Japanese patients compared with US patients (4.3% versus 26.7%, *p* < 0.0001) [7] and among Taiwanese patients compared with patients from the rest of the world (3.3% vs. 19.5%, *p* < 0.001) [47].

Complement inhibitors are a mainstay of treatment for patients with PNH [57]. Clinical trials and observational studies support the overall effectiveness and safety of eculizumab and ravulizumab in Asian patients [37,58,59,60,61,62,63,64]. Long-term follow-up of three international clinical trials of eculizumab (n = 195) found that the proportion of patients free of TEs increased from 67.7% before eculizumab initiation to 96.4% during eculizumab treatment [27]. Results of Asian studies appear to be generally consistent with these international data. No TE events were reported during eculizumab treatment of up to 2 years duration in the AEGIS clinical trial in Japan (n = 27) [37,58]. In a Korean study, TE resolved during treatment in all 19 patients who had TE at the time of eculizumab initiation, although it recurred in one patient [60]. A nationwide Korea study found no significant difference in the incidence of new venous or arterial thrombosis between eculizumab-treated patients with severe PNH and eculizumab-untreated patients with less severe PNH [61]. Analysis of real-world data from Korea (n = 80) found that TE resolved during eculizumab treatment in 70% (14/20) of patients who had TE present before eculizumab initiation [64]. Data regarding the use of C5 inhibitors specifically in the Asian pediatric population with PNH are lacking.

Although the utility of C5 inhibition to mitigate the risk of complications such as TE in PNH patients is widely accepted in Asia, access to complement inhibitors varies among countries. In Japan, anti-complement therapy is indicated for patients with severe PNH (including those with a current or past history of thrombosis, those requiring regular blood transfusions for hemolysis, and those with renal impairment) and for some patients with moderate PNH (e.g., those with moderate renal impairment or smooth muscle spasms) [65]. In South Korea, the cost of C5 inhibitor therapy is supported by the National Health Insurance Service only for patients who meet all of the following criteria: PNH granulocyte count ≥10%; LDH ≥ 1.5 times the upper limit of normal (ULN); transfusion-dependent anemia (≥4 units of red blood cells in the past 12 months); and presence of ≥1 complication (thrombosis, renal insufficiency, pulmonary insufficiency, and recurrent smooth muscle spasm) [61]. In Hong Kong and Taiwan, the use of complement inhibitors in PNH is limited in the absence of complications such as TE or other high-risk characteristics, such as severe anemia caused by hemolysis or renal impairment [66]. Restricting the use of complement inhibitors to high-risk patients only has clinical implications, as non-treated patients with PNH remain at increased risk of TE.

## 4. Biomarkers/Risk Factors for TE in Asian PNH Patients

International datasets have reported an association between larger granulocyte clone size (especially >50%) and a greater risk of TE [42,46,67,68,69,70], although TE can still occur in patients with small clone sizes (<10%) [42,67]. Conversely, a Korean Registry study found no correlation between clone size and risk of TE [38]. Independent predictors of TE in Korean patients were LDH ≥ 1.5 × ULN, pain, and male sex (Table 2) [38,56]. The predominant risk factor was LDH ≥ 1.5 × ULN at diagnosis (reflecting hemolysis), increasing the odds of TE by 7- to 12-fold relative to a lower LDH level. The presence of abdominal pain, chest pain, dyspnea, or hemoglobinuria further increased the risk compared with elevated LDH alone [38].

An International PNH Registry analysis identified a history of TE, ≥30% GPI-negative granulocytes, and LDH ≥ 1.5 × ULN plus ≥2 high disease-activity criteria as independent risk factors for TE, while abdominal pain was a possible predictive factor in univariate analysis [69]. French investigators identified age > 55 years, use of transfusions, and thrombosis at the time of PNH diagnosis as independent risk factors for thrombosis [71]. Pregnancy and the postpartum period are also recognized as being associated with a high risk of TE among PNH patients [72,73,74].

## 5. Monitoring of TE in Asian PNH Patients

Although data for monitoring of TE specific to Asian PNH patients are limited, reference can be made to clinical evidence in non-Asian populations, giving due consideration to the lower level of intrinsic thrombogenicity in Asians compared with non-Asians. Mutations in factor V (Leiden mutation) or prothrombin G20210A, which are risk factors for venous TE in Caucasians, are virtually absent in East Asian populations [75]. Japanese guidelines report an incidence of pulmonary TE per million population at around 62 cases in Japan compared with 500 cases in the US [76]. Racial background would be expected to impact on thrombogenicity potential also in patients with PNH.

Monitoring for signs and symptoms of TE in patients with PNH must factor in any past history of TE, current symptoms (e.g., abdominal pain, chest pain, dyspnea, or neurological symptoms) that may suggest TE in typical or atypical sites, and laboratory test results (e.g., D-dimer and platelet count) [77]. Periodic monitoring for D-dimer and fibrin degradation products in PNH patients with an elevated serum LDH level may be useful for detecting subclinical clots. Patients who develop signs or symptoms suggestive of TE should be evaluated using the most appropriate investigation for site and presentation [78]. Particular attention should be given to signs and symptoms of arterial thrombosis in view of the higher relative risk in Asians than non-Asians [79] and its propensity to occur at unusual sites, e.g., the portal system. Patients should be educated about TE symptoms to facilitate prompt seeking of medical attention.

Specific markers of TE risk in PNH are lacking. Clone size on its own is not a reliable estimate of TE risk [80]. However, the positive association between larger clone size and increased procoagulant factor activity translates into increased thrombin and fibrin formation, which is detectable by higher D-dimer levels in PNH patients compared with controls [15,81]. Studies of eculizumab have shown that D-dimer levels are increased in PNH patients before treatment and decrease during treatment [24,25,36]. Although D-dimer is not specific to PNH [82], it may nevertheless serve as a useful marker of hemostatic activation/thrombotic risk in patients with PNH [80,83]. Assessment of D-dimer levels during routine follow-up should be considered for PNH patients, including those receiving complement inhibitors, especially if they have a history of TE [77,83]. Levels should decrease rapidly after the start of C5 inhibitor therapy and remain suppressed [24,83]. In patients who are not candidates for C5 inhibitors, persistently elevated D-dimer levels may signal the need for prophylactic anticoagulation [84].

## 6. Anticoagulant Therapy in Asian PNH Patients

As data for anticoagulant use specific to Asian patients with PNH are limited, a general approach in Asia is to follow guidance from the international literature.

Any decisions about anticoagulant therapy in patients with PNH must balance a reduction in TE risk against a potential increased risk of bleeding [67], as recognized with warfarin [67,68]. Lower baseline rates of TE in Asians than non-Asians [85] and higher bleeding rates in Asians while taking warfarin [75,86,87] underscore the need for a considered approach. In Hong Kong, for example, PNH patients without TE do not receive primary prophylaxis. PNH patients with TE commence anticoagulant therapy, and TE is an indication for anti-complement therapy. A decision to continue anticoagulant therapy on a long-term basis after starting anti-complement therapy is taken on a case-by-case basis.

For PNH patients with no history of TE who are receiving a C5 inhibitor, adding anticoagulant therapy for primary prophylaxis is not necessary because of the preventive effect of C5 inhibition [10,78,83]. Where C5 inhibitors are not available, primary prophylaxis with warfarin may be considered for patients who have a substantial PNH clone, platelet count > 100 × 10^9^/L, and no known risk factor for hemorrhage [10,67,84], although anticoagulation does not eliminate the risk of TE completely [5]. Some expert groups recommend anticoagulation as primary prophylaxis for patients who do not meet eligibility criteria for eculizumab treatment [83,88], whereas others suggest that anticoagulation should be reserved for certain high-risk patients, such as those with persistently elevated D-dimer levels or who are pregnant [84].

All patients who experience an acute TE event should receive anticoagulation [10,80,89]. C5 inhibitor therapy should also be started immediately (if the patient is not already on treatment), as anticoagulation alone may not provide sufficient long-term control [10,78,80,83,89].

At present, it is unclear whether anticoagulant therapy should be continued alongside C5 inhibition as secondary prophylaxis after a TE event [90]. Some experts recommend continuing anticoagulation indefinitely unless there is a contraindication [5,78,83]. Others advocate, in the presence of C5 inhibitor therapy, to discontinue anticoagulation after 3–6 months of concomitant therapy, provided that the TE has fully resolved, PNH is well controlled, and there are no persistent risk factors for TE [89,90].

The need for anticoagulant therapy can be summarized according to a 2 × 2 concept incorporating TE history and complement inhibitor treatment (Table 3).

In terms of treatment selection, heparins and coumarins can be used both prophylactically and therapeutically [65,88]. Heparin or low-molecular-weight heparin (LMWH) is generally recommended immediately after an acute TE event, with heparin being recommended in Japan as LMWH is not covered by health insurance for the treatment of PNH [10,65,89]. Although warfarin is generally used for long-term prophylaxis [10,65], some experts advocate continued use of LMWH to facilitate the management of any bleeding [92]. Direct oral anticoagulants (DOACs) may be effective but are not well studied in PNH [5,65,89]. Compared with coumarins, DOACs have the advantages of fewer food and drug interactions and do not require regular monitoring.

### Special Situations

As thrombocytopenia increases the risk of bleeding and may be associated with serious hemorrhage during anticoagulant therapy [10,92], its presence is regarded as a relative contraindication to anticoagulation in patients with PNH [10]. While specific evidence-based criteria contraindicating anticoagulant therapy based on platelet counts in PNH patients are limited, a guideline suggests that anticoagulation is generally contraindicated when the platelet count is below 10 × 10^9^/L due to an increased risk of bleeding [93]. Additionally, patients with platelet counts below 100 × 10^9^/L have demonstrated a lower odds ratio for thromboembolic events (OR, 0.43; 95% CI, 0.22–0.85; *p* = 0.0152), indicating a reduced risk of thrombosis as platelet counts decline. Therefore, the decision to initiate anticoagulation in PNH patients with thrombocytopenia should be individualized, carefully weighing the risks of thrombosis against the potential for bleeding [94].

Pregnancy increases TE risk in PNH patients by exacerbating the prothrombotic state and increasing complement activation [95]. In the pre-eculizumab era, pregnancy was associated with substantial TE-related maternal and fetal morbidity and mortality [72,74,96]. Available evidence indicates that eculizumab is safe and effective for managing PNH during pregnancy and reduces the risk of TE and associated adverse maternal and fetal outcomes [95,96,97,98]. Guidelines recommend anticoagulation in pregnant patients, particularly those with a history of TE or who are otherwise at high risk, although it should also be considered in patients without a history of TE [65,83]. As most maternal TE events occur during the postpartum period, anticoagulant therapy should be continued for 6 weeks after delivery [65,83]. LMWH is generally used [83,89,95,96,97]. In Japan, unfractionated heparin is recommended as LMWH is not approved for TE prophylaxis in PNH [65].

Budd–Chiari syndrome due to hepatic vein thrombosis can occur in patients with PNH despite anticoagulant prophylaxis [10]. Anticoagulation will not necessarily restore adequate hepatic blood flow, and despite the risk of hemorrhage, some patients may require thrombolytic therapy [10,88,99]. Eculizumab (or other C5 inhibitor) can reduce the risk of new thrombosis and facilitate clot lysis in patients with Budd–Chiari syndrome and, as such, may reduce the need for thrombolytic therapy [100,101,102,103].

## 7. Challenges and Opportunities

One of the main challenges of managing TE in Asian patients with PNH is the lack of published evidence specific to the region. This situation needs to be addressed in view of the genetic and pharmacological differences between Asian and non-Asian populations. Additional observational studies, particularly analyses of international and regional registries, would provide valuable epidemiological information about the TE rate for Asian and non-Asian patients, including pediatric patients. The possibility of regional collaboration in clinical trials and observational studies could be explored as a means of obtaining data from a larger number of Asian patients.

Despite a potentially lower risk of TE in Asian than non-Asian patients, physicians must remain alert and ensure that PNH patients are monitored regularly for signs and symptoms suggestive of TE. Regular review can also identify any changes in general disease characteristics or risk profile so that treatment can be adjusted as necessary. Assessing TE risk in PNH patients is complicated by the presence of confounding factors such as cardiovascular disease or other comorbidities. All patients with PNH should be assessed carefully for other risk factors for TE and, if identified, managed appropriately in a multidisciplinary environment.

A specific challenge when managing TE risk in Asian patients with PNH is that the most appropriate therapy for arterial thrombosis (anticoagulant and/or antiplatelet) has yet to be defined. This knowledge gap must be addressed, given the higher incidence of arterial thrombosis as the cause of TE events in Asian populations.

More studies in Asian populations are needed to determine the risk–benefit ratio of primary prophylaxis with anticoagulants. The risk–benefit ratio of long-term anticoagulation (after initial therapy) in patients with TE who are receiving anti-complement therapy must also be further evaluated. The role of DOACs versus warfarin in patients with PNH is not well reported and warrants further study.

C5 inhibitors have significantly reduced TE risk in PNH patients. At present, data are emerging for proximal complement inhibitors, including pegcetacoplan (C3 inhibitor), danicopan (factor D inhibitor), and iptacopan (factor B inhibitor). Danicopan, which is used in combination with eculizumab or ravulizumab, may retain an ability to reduce TE risk similar to that of C5 inhibitors, although this has yet to be confirmed. Pegcetacoplan and iptacopan have demonstrated an ability to effectively manage anemia in patients with PNH while maintaining LDH levels comparable to those seen with C5 inhibitors [104,105]. However, in clinical trials, both agents have shown a risk, albeit infrequent, of significant breakthrough hemolysis [106,107,108]. The long-term clinical consequences of intravascular hemolysis episodes in real-world settings remain unclear. Data specific for Asian populations are limited to a subgroup analysis of the phase 3 PEGASUS study of pegcetacoplan, in which no thrombotic events occurred in 10 Japanese patients treated with pegcetacoplan (n = 5) or eculizumab (n = 5) [109].

Another general management challenge in PNH is to identify methods of improving patients’ compliance with long-term treatment in order to reduce the risk of TE and other disease complications. Education is likely to be key in this regard, ensuring that patients understand the benefit–risk balance associated with continuing or discontinuing treatment, including the risk of TE. Adherence is especially important with proximal complement inhibitors (e.g., pegcetacoplan and iptacopan) used as monotherapy because substantial improvements in hemoglobin levels paradoxically increase the risk of severe breakthrough hemolysis. Patients should be reminded regularly to not miss doses when taking oral medications such as iptacopan. Patients should also be educated about the symptoms of TE, including those affecting atypical sites, so that medical attention can be sought in a timely manner.

Complement inhibitors are arguably the most important therapy for TE in PNH. However, access to and reimbursement of anti-complement therapy after a TE event is limited in lower-income countries. Increased availability of anti-complement therapies—for instance, through the use of biosimilar products—may lower the cost of treatment and facilitate access for a broader range of patients (e.g., those not covered by insurance), potentially contributing to a reduced incidence of associated TE.

## 8. Conclusions

TE is a major cause of morbidity and mortality in patients with PNH. Although treatment with complement inhibitors greatly reduces the risk of TE, events requiring anticoagulation may still occur. Irrespective of the lower risk of TE in Asian than non-Asian PNH patients, physicians and patients must be aware of the possibility and remain alert for associated signs and symptoms. Prompt and appropriate treatment is vital to prevent adverse outcomes.

## Figures and Tables

**Table 1:** Comparison of thrombotic characteristics between Asian and non-Asian patients as reported in a meta-analysis of six retrospective studies [8] and an analysis of the International PNH Registry [9].

Characteristic	Reference	Asian	Non-Asian	*p*-Value
Incidence rate	[8] ^a^	11.5%	32.5%	0.000
History of TE	[9] ^b^	3.6%	8.9%	<0.01
Type of TE, frequency	[9] ^b^			
Venous		3.1%	7.3%	0.02
Arterial		0.4%	2.1%	0.09
Sites of thrombosis, proportion in all TE events	[8] ^a^			
Venous (abdominal)		35.1%	50.5%	0.024
Arterial		23.0%	1.1%	0.000
Death rate	[8] ^a^	19.4%	19.4%	0.968
Death from TE	[8] ^a^	6.9%	43.7%	0.000
Use of anticoagulant therapy ^c^	[9] ^b^	8.5%	16.2%	0.002

^a^ Meta-analysis comparing clinical characteristics of patients with PNH from Japan, Korea, and China (n = 866), and France, the UK, and the US (n = 799). ^b^ Analysis of clinical characteristics between Asian patients (n = 246) and non-Asian patients (n = 1547) enrolled in the International PNH Registry. ^c^ Heparin, warfarin, etc. PNH, paroxysmal nocturnal hemoglobinuria; TE, thromboembolism.

**Table 2:** Independent predictors for thromboembolism in patients enrolled in the Korean PNH Registry [38,56].

Parameter	Odds Ratio (95% CI)	*p*-Value
LDH ≥ 1.5 × ULN	7.0 (1.5–32) ^a^ 12.21 (1.61–92.86) ^b^	0.013 0.016
Male sex	2.19 (1.02–4.72) ^b^	0.045
Pain	2.79 (1.09–7.17) ^b^	0.033
LDH ≥ 1.5 × ULN plus abdominal pain	17.79 (2.33–36.01) ^a^	0.006
LDH ≥ 1.5 × ULN plus chest pain	19.04 (3.74–96.99) ^a^	<0.001
LDH ≥ 1.5 × ULN plus dyspnea	10.35 (2.31–46.45) ^a^	0.002
LDH ≥ 1.5 × ULN plus hemoglobinuria	10.28 (1.34–79.02) ^a^	0.025

^a^ Based on analysis of 301 patients. ^b^ Based on analysis of 217 patients. CI, confidence interval; LDH, lactate dehydrogenase; PNH, paroxysmal nocturnal hemoglobinuria; ULN, upper limit of normal.

**Table 3:** Anticoagulant therapy in Asian patients with PNH: 2 × 2 concept.

TE History	Complement Inhibitor	
	Yes	No
Yes	**Administer anticoagulant therapy** Patients already receiving a complement inhibitor who experience an acute TE should be given anticoagulation in addition [10,80,89]. Consider discontinuing anticoagulation after 3–6 months of concomitant therapy provided that the TE has fully resolved, PNH is well controlled, and there are no persistent risk factors for TE [89,90].	**Administer anticoagulant therapy** Patients not receiving a complement inhibitor who experience an acute TE should be given anticoagulation and commence treatment with a complement inhibitor [10,78,80,83,89]. In settings where complement inhibitors are not available, anticoagulation should be continued for secondary prophylaxis.
No	**No anticoagulant therapy** Patients without a history of TE who are receiving a complement inhibitor do not need to receive anticoagulation as primary prophylaxis [10,78]. As anticoagulant therapy can be dangerous in patients with low platelet counts, prophylactic anticoagulation should not be initiated in patients without a history of TE who do not meet the eligibility criteria for complement inhibitor treatment [91].	**Consider anticoagulant therapy** Prophylactic anticoagulation can be considered in patients with persistently elevated D-dimer levels, pregnant women, and during preoperative periods [84,88,91]. In settings where complement inhibitors are not available, consider anticoagulant primary prophylaxis for patients with a substantial PNH clone, platelet count × 10^9^/L, and no known risk factor for hemorrhage [10,67,84]. Primary prophylactic anticoagulation should also be considered in patients who do not respond to complement inhibitors [88].

PNH, paroxysmal nocturnal hemoglobinuria; TE, thromboembolism.

## Data Availability

This guidance review and its supporting references will be available on NCBI (PubMed).
